# Fall prevention education among older hospital inpatients: a systematic review

**DOI:** 10.3389/fpubh.2026.1829617

**Published:** 2026-07-08

**Authors:** Yanzhi Yi, Yabin Wen, Olivia Monteiro, Xuelan Wu, Lawrence T. Lam

**Affiliations:** Faculty of Medicine, Macau University of Science and Technology, Macau, Macao SAR, China

**Keywords:** education, efficacy, older inpatients, fall prevention, systematic review

## Abstract

**Background:**

Hospitalized older adults are at high risk of falls. Although fall prevention education is recommended in hospital settings, the efficacy of different education programs remains inconsistent. This study aimed to systematically review the effectiveness of fall prevention education interventions among older inpatients.

**Methods:**

We conducted a systematic review of randomized or trial-linked evaluations of fall prevention education programs for inpatients aged ≥60 years. A comprehensive search was performed across PubMed, Embase, Web of Science, Scopus, and Cochrane Library databases from inception to January 7, 2026. Risk of bias was assessed for all included evaluations. Intervention characteristics, including format, content, duration, providers, and theoretical frameworks, were extracted and analyzed.

**Results:**

Twelve reports describing 11 trial-based evaluations were included, with risk of bias ranging from low to high. Most reports described session-level or initial education contact of ≤1 h, whereas six reports incorporated protocol-specified repeated education, reinforcement, or post-education contact. Interventions varied considerably in format, content, duration, and providers, including physiotherapists, an occupational therapist, nurses or nurse investigators, a trained MSc sport science student, allied health assistants, and investigators with unspecified professional background. Five evaluations reported reductions in at least one fall-related measure, and one evaluation reported benefit only among cognitively intact patients. Six evaluations reported favorable education-related outcomes where measured. Behavior-related outcomes were mixed: three reports showed improvements in selected risk-reducing behaviors, whereas two reports found no significant behavior-related between-group differences. The 11 assessed programs used several theoretical or design approaches and demonstrated high educational-design quality, with scores ranging from 15 to 17 on a 17-point scale.

**Conclusion:**

Fall prevention education may help reduce falls and improve related outcomes among older inpatients; however, substantial heterogeneity in study design, intervention characteristics, and outcome measures limits generalizability. The use of explicit theoretical or design approaches may support more structured educational intervention design, but further comparative evaluation is needed to determine which delivery models and components are most effective.

**Systematic review registration:**

https://www.crd.york.ac.uk/PROSPERO/view/CRD420251238711.

## Highlights

### What is already known?

- Fall prevention education is recommended for hospitalized older adults at risk of falls.
- Individualized or tailored education has been evaluated as a strategy to improve fall-risk awareness, engagement in prevention strategies, and fall-related outcomes.
- The effects of fall prevention education on fall-event outcomes have been inconsistently reported.

### What this paper adds?

- Trial-based evidence on theory-informed fall prevention education for hospitalized older adults remains limited and heterogeneous.
- The Health Belief Model was the most frequently used theoretical framework among the included fall prevention education programs.
- Evidence remains limited on how theoretical constructs, education content, and delivery features translate into sustained behaviour change and fall-event reduction.

## 1 Introduction

A fall is an event that occurs when a person inadvertently encounters the floor, ground, or another lower level during displacement. Falls are a significant public health concern worldwide. It is estimated that 684,000 fatal falls occur globally each year, representing the second leading cause of unintentional injury deaths. Although the majority of falls are not deadly, falls account for approximately 37.3 million annual incidents that are serious enough to require medical attention. Globally, more than 38 million disability-adjusted life years (DALYs) are lost each year because of falls, resulting in more DALYs lost than traffic injuries, drowning, burns, and poisoning combined (1). Older adults are at the greatest risk of death or serious injury from falls, with the risk increasing with age. The mortality rate in cases of fatal falls is highest among people over 60. In the United States, 20–30% of older adults who fall suffer moderate to severe injuries, including bruises, hip fractures, and head trauma. The financial costs of fall-related injuries are considerable, with average health system costs per fall injury for people aged 65 years or older being $3,611 and $1,049 in Finland and Australia, respectively (1).

Inpatient fall rates, typically measured per 1,000 bed days, generally fall within the range of 2 to 8 in acute hospitals, geriatric wards, and emergency settings (2, 3). However, among older inpatients, studies have shown a notably higher incidence of falls, reaching 12.6 per 1,000 bed days (4). Hill observed that in eight rehabilitation and geriatric wards in Australia, there were 10.9 patient falls per 1,000 bed days (5). In 2004, a significant incidence of 18.0 falls per 1,000 bed days was documented in a geriatric care unit at a regional general hospital in the United Kingdom (6). Two systematic reviews have shown that risk factors for falls in hospitalized patients include a history of falls, use of walking aids, and disability, advanced age, cognitive impairment, the use of sedatives and antidepressants, gait instability, agitated psychosis, and urinary incontinence (7, 8). Advanced age is a prominent intrinsic risk factor for inpatient falls, with fall-related injury incidence rates spanning from 6.8 to 72.1% (9). As a result, hospitalized older patients, particularly those with a fall history, are frequently at an increased risk of experiencing falls during their hospital stay.

Strategies for preventing inpatient falls encompass patient and staff education, environmental enhancements, assistive devices, therapeutic exercise, medication review, nutritional interventions, cognitive-care protocols, and continuous multidisciplinary quality improvement and effective leadership (10–12). The World Guidelines strongly recommend tailored fall prevention education for all inpatients aged ≥ 65 years and other high-risk groups (recommendation 1A) (13). Well-designed programs significantly improve older patients’ awareness of fall risk and motivate them to adopt protective behaviors (14). A systematic review indicates that educating patients and staff effectively reduces inpatient falls (11). An updated Cochrane review of fall-prevention interventions in hospitals reported that tailored education probably reduces both the rate of falls and the risk of falling among older hospital patients (12). Hospitalized older adults are at higher risk of falling and often sustain more severe injuries, resulting in prolonged length of stay and increased medical and financial burden. Health education can mitigate this problem by raising awareness of fall risk and providing specific prevention strategies during hospitalization (13). Advancing age is accompanied by declining auditory, visual and cognitive function, which reduces the effectiveness of conventional educational interventions and underscores the need for tailored approaches. This systematic review was therefore undertaken to evaluate the efficacy of different fall prevention education methods for older inpatients and to generate evidence-based recommendations for hospital managers.

## 2 Method

### 2.1 Aim

The aims of this review are threefold: (i) to identify existing fall prevention education (FPE) programs and evaluate their efficacy among older inpatients; (ii) to identify the theoretical models underlying the educational designs utilized in these programs; (iii) to assess the quality of these FPE programs.

### 2.2 Design

This systematic review was conducted in accordance with the Preferred Reporting Items for Systematic Reviews and Meta-Analyses (PRISMA) guidelines (15). The protocol was registered with the PROSPERO database on 27 November 2025 (registration number: CRD420251238711).

### 2.3 Inclusion and exclusion criteria

The PICO framework guided the research question: (p) hospital inpatients aged 60 years or older; (i) fall prevention education (FPE); (c) usual care or alternative education formats; (o) fall prevention-related outcomes, including fall-related, education-related, and behavior-related outcomes.

Inclusion criteria are: (a) studies with a target population aged 60 years or above; (b) participants are hospital inpatients; (c) research studies on fall prevention education (FPE); (d) studies using an individually randomized or cluster-randomized controlled trial design, or reports providing additional data linked to an eligible randomized controlled trial (16). Exclusion criteria are: (a) studies on outpatients; (b) studies lacking a definitive outcome for evaluating the efficacy of a fall prevention education program; (c) interventions where education is only a part of the program rather than the sole component; (d) abstracts, reports, conference posters, or non-English publications.

### 2.4 Search strategy

Five electronic databases (PubMed, Embase, Web of Science, Scopus, and Cochrane Library) were searched from their inception to January 07, 2026. Two researchers (YW and XW) searched independently and concurrently using the consistent strategy. Different strategies were employed to achieve comprehensive search, tailored to the characteristics of each database. Details of the search strategies were reported in the Appendix I.

### 2.5 Search selection

Study selection followed the PRISMA 2020 flow diagram and was conducted independently by two reviewers (YW, XW) in two sequential steps (15):

- Title/abstract screening: all retrieved records were screened to exclude obviously ineligible studies; potentially relevant records were advanced to full-text review.
- Full-text assessment: potentially eligible studies were evaluated against predefined inclusion and exclusion criteria. Discrepancies were resolved by discussion or, if necessary, by adjudication of the corresponding author.

**Figure 1:**
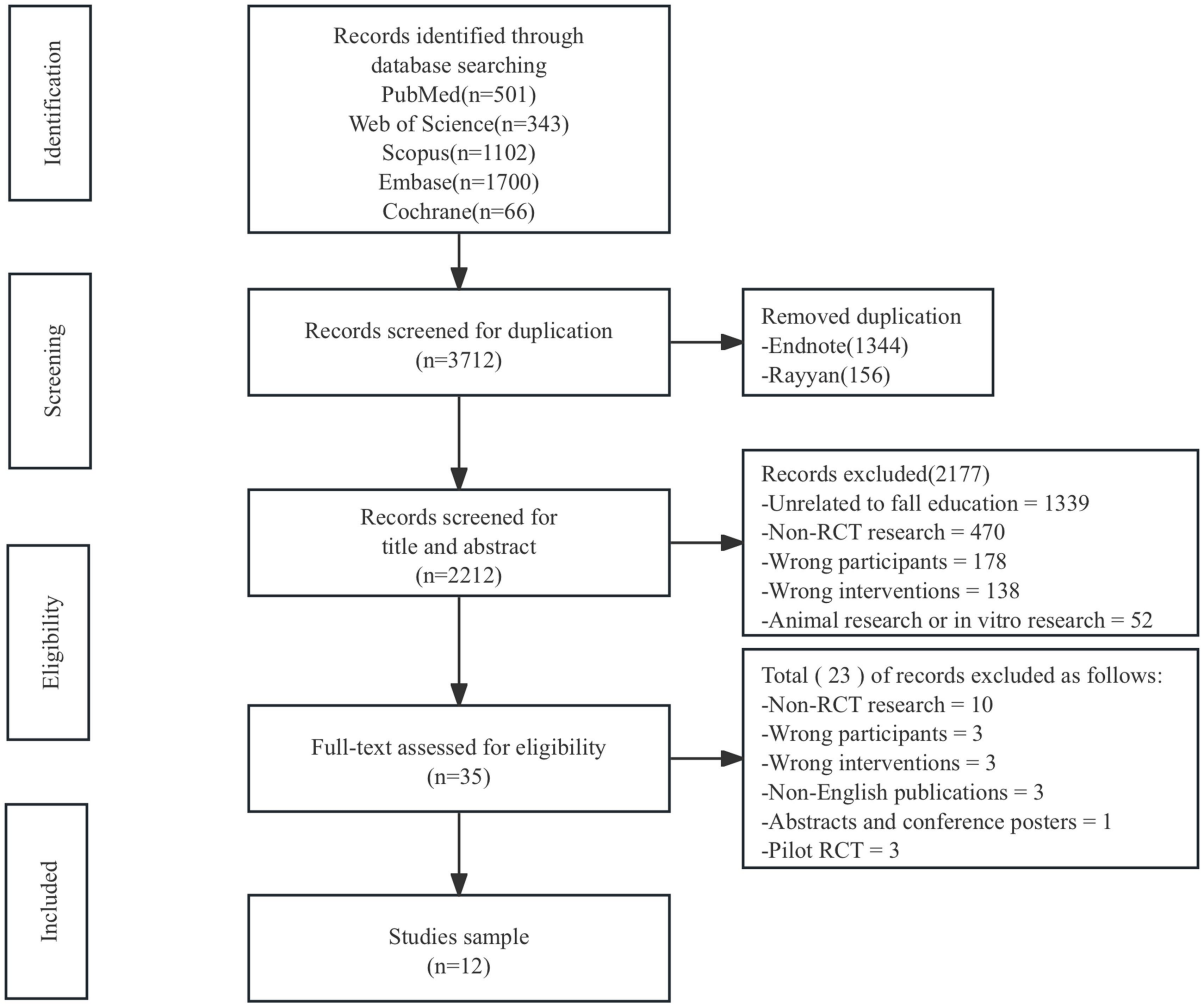
The PRISMA flow diagram.

After full-text assessment, 12 reports describing 11 trial-based evaluations were included in the review. The detailed selection process and reasons for full-text exclusion are presented in (Figure 1).

### 2.6 Data extraction

Two researchers (Y. Y and Y. W) independently extracted data from the included reports and entered the data into a standardized extraction table. The extracted characteristics included participants, interventions, comparators, follow-up durations, outcomes measures main findings, and other relevant details, which are summarized in Table 1. Outcomes were extracted as reported and grouped narratively into fall-related outcomes, education-related outcomes, behavior-related outcomes, and, where reported, related health-service outcomes such as readmission and length of stay.

**Table 1:** The characteristics of educational programs.

Study/Author	Haines et al. (14)	Hill et al. (20)	Haines et al. (23)	Hill et al. (21)	Hill et al. (5)	Hill et al. (25)/Naseri et al. (24)
Primary citation/Relationship to parent trial	Education-recommended subgroup (14) of Haines targeted multifactorial RCT (30)	DVD–workbook RCT with an additional no-education quasi-control group (20)	Independent 3-arm RCT (23)	Pilot RCT (21)	Pragmatic stepped-wedge cluster RCT (5)	Hill 2019: Primary RCT (25) Naseri 2019: same-RCT process/behavior evaluation (24)
Sample/Losses	226 (IG 115, CG 111)/0	100 randomized (DVD 51, workbook 49); quasi-control 122/0	1,206 (CPG 401, MG 424, CG 381)/0	50 (IG 25, CG 25)/2%	3,606 admissions (IP 1623, CP 1983)/0	390 randomized (IG 196, CG 194); Hill: 382 (IG 194, CG 188)/2%; Naseri: 292 (IG 149, CG 143)/23.6%.
Age (years)	IG: 83 (77, 88) CG: 82 (75, 86)	DVD: 74.5 ± 9.7 Workbook: 76.1 ± 10.5 Quasi-control: 79.3 ± 8.3	CPG: 75.3 ± 11.0 MG: 74.7 ± 11.7 CG: 75.3 ± 10.1	IG: 78.2 ± 9.0 CG: 78.3 ± 7.5	IP: 81.4 ± 9.3 CP: 82.1 ± 8.3	IG: 77.4 ± 8.8 CG: 78.1 ± 8.5
Locations/Settings	Subacute wards of the Peter James Center, a metropolitan aged-care hospital in Melbourne, Australia.	Geriatric, medical, and orthopedic wards in Perth and Brisbane, Australia; the restorative unit or medical ward of the Swan Districts Hospital (SDH), Perth, Australia.	Acute and subacute wards of Princess Alexandra Hospital, Brisbane, and Swan Districts Hospital, Perth, Australia.	Stroke and rehabilitation units of Swan Kalamunda Health Service, Australia	Eight rehabilitation units in general hospitals, Australia.	Rehabilitation wards at three hospitals in Perth, Western Australia.
Prevention target	In-hospital falls	In-hospital fall-risk awareness/knowledge	In-hospital falls	Post-discharge falls	In-hospital falls	Post-discharge/home falls
Education setting	Bedside education	Bedside education	Bedside education	Bedside education (pre-discharge) + telephone reinforcement	Ward-level bedside education	Bedside education (pre-discharge) + post-discharge telephone reinforcement
Intervention	Usual care + one-to-one bedside education; other targeted components if indicated	DVD-based fall prevention education	Usual care + written and video-based materials + 1-to-1 follow-up	Usual care + written and video materials + individual tailored discussion sessions + one follow-up telephone call	Usual care + multimedia patient education package (DVD + written workbook) with individually tailored follow-up sessions	Hill 2019: Usual care + individualized multimodal fall prevention education, including video/workbook, therapist-led structured discussion, and goal/action planning. Naseri 2019: same intervention; process report emphasizing personalized discussion, preference-based post-discharge goals, and coaching calls.
Comparator	Usual care; recommended education not delivered	Workbook; Usual care	MG: usual care + written and video-based materials CG: usual care	Usual care	Usual care	Usual care + 45 min positive aging social education.
Individual/Group delivery	Individual/one-to-one	Individual	Individual/one-to-one	Individual/one-to-one	Individual/ward-level delivery	Individual/one-to-one
Education content	Falls risk factors, the nature of falls, general mechanisms of falls, steps to prevent falls, participant quizzes, goal setting and review.	Risk of falls and fall-related harm; Fall prevention strategies; Foster patient belief; Cue for action/planning of fall prevention strategies.	Epidemiologic falls data, causes of falls, self-reflection of individual risk, problem-area identification, development of preventive strategies and behaviors, goal setting, and goal review.	Seeking assistance for functional activities; Gradually resuming functional activities; Planning to participate in an exercise program. The risk of falls and functional decline after discharge and about fall prevention strategies to undertake in the period after discharge.	Personal fall risk, falls epidemiology and prevention, benefits of engaging in fall prevention activities, cues to action, self-efficacy, goal setting and action planning.	Hill 2019: seeking assistance with personal care/daily activities; exercise to regain functional mobility; gradual return to usual activities and home modifications. Naseri 2019: personalized strategies for ADL/IADL assistance, home-hazard modification, and safe exercise.
Mode of delivery	Face-to-face bedside discussion; booklet.	DVD, written workbook	Video and written materials; 1-to-1 bedside follow-up	Written and video materials, individual tailored discussion sessions, and one telephone call	DVD; workbook; individually tailored follow-up session.	Bedside workbook/video education, therapist-led structured discussion, individualized action plan, and monthly telephone reinforcement.
Education session duration	15–35 min per session	DVD 14 min; workbook 24 pages; up to 1 h to study materials	Median 25 min (IQR 20–36)	45 min	Median 45 min (IQR 35–55)	≈45 min total in hospital; telephone calls ≈60 min total
Education frequency/follow-up	Twice weekly; median 4 sessions (IQR 2–5)	One-off education; no reinforcement	1-to-1 bedside follow-up during the first study week; number of sessions at physiotherapist discretion	Two bedside sessions (actual 2–5); one telephone call 2 weeks after discharge	Median 2 sessions (IQR 1–3); ward visits 2–3 times/week	2–4 pre-discharge sessions; 3 monthly telephone reinforcement calls after discharge
Educational design principles and models	Threat appraisal, Protection Motivation Theory, and goal setting/review.	Health Belief Model; recommended patient-education material principles.	Health Belief Model	Health Belief Model; adult learning principles	Health Belief Model; adult learning principles; health behavior-change principles	COM-B model of behavioral change theory.
Education provider	Occupational therapist	Investigators	Trained physiotherapist	Trained physiotherapist	Trained physiotherapist	Experienced physiotherapist
Main results	Significantly lower incidence of falls in the intervention group (8.2 falls/1000 participant-days vs. 16.0 falls/1000 participant-days, log-rank test: *p* = 0.007). The difference in the proportion of fallers was not significant (RR = 1.21, 95% CI: 0.68–2.14).	DVD group showed higher self-perceived risk of falling (*p* = 0.04), confidence (*p* = 0.03) and motivation (*p* = 0.04) than workbook group. DVD group and workbook group gave more “desired” responses across knowledge items than quasi-control (*p* < 0.001).	Falls per 1,000 patient-days did not differ significantly across the total sample (CG 9.27; MG 8.61; CPG 7.63). There was a significant interaction between intervention and cognitive impairment. Among cognitively intact patients, falls were fewer in the CPG than in the MG and CG (4.01 vs. 8.18 and 8.72 falls/1000 patient-days; CPG vs. MG: aHR 0.51, 95% CI 0.28–0.93; CPG vs. CG: aHR 0.43, 95% CI 0.24–0.78). Among cognitively impaired patients, injurious falls were higher in the CPG than in the CG (7.49 vs. 2.89 injurious falls/1000 patient-days).	Participants in the IG were significantly more likely to plan how to safely restart functional activities than those in the CG (AOR 3.80, 95% CI 1.07–13.52; *p* = 0.04), and were more likely to complete their own home exercise program (AOR 2.76, 95% CI 0.72–10.50; *p* = 0.14). IG was significantly more knowledgeable, confident and motivated to engage in fall prevention strategies after receiving the education than the CG. Fall rate was lower in the IG than CG (5.4 vs. 18.7 falls/1000 person-days; *p* = 0.05).	Fewer falls were observed in the intervention period than in the CP (7.80 vs. 13.78 falls/1000 patient-days; ARR 0.60, 95% CI 0.42–0.94; *p* = 0.003). Injurious falls and fallers were also reduced in the IP (injurious falls: 2.63 vs. 4.75/1000 patient-days; ARR 0.65, 95% CI 0.42–0.88; *p* = 0.006; fallers: 8.38% vs. 12.51%; adjusted OR 0.55, 95% CI 0.38–0.81; *p* = 0.003). There was no significant difference in length of stay.	Hill 2019: No significant differences between groups in 6-month fall rate, injurious fall rate, or faller proportion after discharge. Naseri 2019: No significant differences between groups in engagement in fall prevention strategies, including IADL assistance, home modifications, or exercise.
Education-related outcomes	Increased (materials understandable; new information gained)	Increased (fall prevention knowledge; education groups vs. no-education quasi-control)	Not mentioned	Increased (fall prevention knowledge, confidence and motivation; self-perceived fall risk)	Not mentioned	Not mentioned
Behavior related outcomes	Increased (modified their actions to reduce their risk of falling)	Increased (confidence and motivation to engage in self-protective strategies; DVD group)	Increased (seeking/waiting for help; identifying environmental hazards; using assistive devices; wearing safe footwear/clothing; doing more exercise)	Increased (seeking formal/informal assistance for ADL/IADL; safely resuming functional activities; participating in a home exercise program; making informal home modifications)	Not mentioned	Naseri 2019: No significant differences in fall prevention strategy engagement, including ADL/IADL assistance, home-hazard modification, and exercise.
Result measures	Falls per 1,000 participant-days; proportion of fallers; education program evaluation survey.	Custom survey: perceived fall risk, falls epidemiology, fall prevention knowledge, confidence and motivation.	Falls, injurious falls, fractures, falls per 1,000 patient-days, proportion of fallers, and injurious falls per 1,000 patient-days.	Participants’ perceptions of education (awareness, knowledge gain, confidence and motivation); engagement in fall prevention strategies; falls/injurious falls/fallers/fractures; falls per 1,000 person-days; injurious falls per 1,000 person-days; faller percentage; hospital admissions or doctors’ visits.	Falls, injurious falls, fallers, fractures, falls per 1,000 patient-days, injurious falls rate per 1,000 patient-days, fallers percentage, and length of stay.	Hill 2019: falls rate, injurious falls rate, faller proportion, Katz Index, Lawton IADL scale, AQoL-6D. Naseri 2019: engagement in fall prevention strategies, including IADL assistance, home modifications, and exercise.
Outcome follow-up duration	In-hospital only; until discharge	Immediate post-intervention survey; no fall-event follow-up	In-hospital only; until discharge or transfer from study wards	1 month after discharge	In-hospital only; during rehabilitation-unit stay	6 months after hospital discharge.
Major findings	Patient education is an important part of a multiple intervention fall prevention approach for the subacute hospital setting.	Delivery of fall prevention education on a DVD compared to a written workbook is more likely to achieve important changes in parameters likely to affect successful uptake of falls prevention messages in the hospital setting.	Multimedia patient education with trained health professional follow-up reduced falls among patients with intact cognitive function admitted to a range of hospital wards, but no significant effect was observed in the total sample.	Tailored education was positively received by older people, resulted in increased engagement in fall prevention strategies after discharge, and is feasible to deliver to older hospital patients.	Individualized patient education programs combined with staff training and feedback, added to usual care, reduced falls and injurious falls in older patients in rehabilitation hospital units.	Hill 2019: Providing individualized fall prevention education prior to discharge did not reduce falls at home after discharge. Naseri 2019: Tailored education did not increase older adults’ engagement in fall prevention strategies after hospital discharge compared with usual care.

Study/Author	Perrot et al. (29)	Dadgari et al. (27)	DeWalt et al. (28)	Valieiny et al. (26)	Morris et al. (22)
Primary citation/Relationship to parent trial	Primary RCT (29)	Primary RCT (27)	Primary RCT (28)	Primary RCT (26)	Feasibility RCT (22)
Sample/Losses	30 (PAPE 15, PA 15)/16.7%	112 (IG 58, CG 54)/10.7%	83 (SG 38, WEG 45)/7.2%	132 (IG 66, CG 66)/0	541 (IG 271, CG 270)/0
Age (years)	PAPE: 80.23 ± 8.09 PA: 78.92 ± 8.01	IG: 60–69 (40%), 70–79 (34%), ≥80 (26%); CG: 60–69 (34%), 70–79 (32%), ≥80 (34%).	SG: 78.5 ± 7.3 WEG: 78.3 ± 8.9	IG: 67.66 ± 6.99 CG: 72.48 ± 8.83	IG: 81.0 CG: 80.0
Locations/Settings	Rehabilitation healthcare service, France.	Imam Ali Trauma and Triage Center, Bojnourd, Iran.	Medical-surgical inpatient units of a community-based mid-sized teaching hospital, USA.	Wards of Shahid Beheshti Hospital, Qom, Iran.	Two participating 30-bed wards at a rehabilitation hospital, Australia.
Prevention target	Fall risk factors and FOF	Post-discharge recurrent falls	Post-discharge home fall risk	Falls and FOF among hospitalized older adults	In-hospital falls
Education setting	Inpatient rehabilitation group education	Hospital-based discharge education + home visit	Hospital-based simulated home environment education (pre-discharge)	Ward-based education (during hospitalization)	Ward-based education (within 48 h after admission)
Intervention	Physical activity program + four 1-h therapeutic patient education sessions.	Discharge planning program + individualized training and booklet.	Usual care + simulation-based fall prevention education in a simulated home environment.	Simulated video education (SVE) through three videos.	Usual care + additional patient fall prevention education.
Comparator	Physical activity	Usual care	Usual care + written fall prevention education handout	Routine care services	Usual care
Individual/Group delivery	Group	Individual/one-to-one	Individual/one-to-one	Individual/one-to-one	Individual/one-to-one
Education content	Knowledge of a fall and its risk factors. Importance of footwear and food hygiene. Strategies for exercise adherence. Environment and affordances.	Importance of falls and risk factors; Internal and external fall risk factors; Control of extrinsic factors/home environment; Motor skills for daily activities.	Identification of 14 environmental fall hazards; Education on 10 perceived environmental fall hazards using a standardized card; Pet-related fall hazards; Strategies to mitigate home fall risks after discharge.	Importance of fall prevention, fall outcomes/complications, risk factors, fall preventability, and strategies; Specific ward-based fall prevention measures, including wristband colors, bed height, caster brakes/bed rails, call bell use, avoiding sudden out-of-bed movements, walkers/wheelchairs, footwear, lighting, eyeglasses, and other precautions; Ward facilities, including the nursing station, emergency exit, toilets, wall rails, toilet hand grabs, nursing call system, and bed lighting.	Face-to-face discussion within 48 h to develop a goal-oriented action plan using motivational interviewing; Guidance on using the pamphlet and discussion of its content; Brief conversations during admission and a day-5 follow-up about fall risk and fall prevention behaviors.
Mode of delivery	Face-to-face group sessions.	Individual education sessions, booklet, home visit, checklist, and instructional video.	Simulation-based education and nurse-guided discussion.	Simulated video education (SVE)	Face-to-face scripted conversation; educational pamphlet.
Education session duration	1 h per therapeutic patient education session.	30–60 min per session.	Approximately 20 min.	15 min total.	Not mentioned.
Education frequency/follow-up	4 sessions, every 2 weeks	4 sessions, including 1 home visit	One-off pre-discharge education	Three videos; available for repeated viewing	Initial education within 48 h, followed by brief conversations during admission and a scheduled day-5 follow-up
Educational design principles and models	Therapeutic patient education	Nursing discharge planning	Simulation-based education	Simulation-based video education	Motivational Interviewing
Education provider	Trained MSc sport science student	Nurse	Nurse investigator	Nurse	Allied health assistants
Main results	Both PA and PAPE groups improved from pre- to post-test on TUG, Tinetti, and FES-I; only FES-I showed a significant group×time interaction, indicating a greater reduction in fear of falling in the PAPE group.	Significant decreases were observed in the IG compared with the CG for falls (*p* < 0.049), readmission (*p* < 0.014), length of hospital stay (*p* < 0.018), and injury severity (*p* < 0.001).	Simulation education had higher fall-risk post-assessment scores than the WEG (*p* = 0.022). Changes in fall-risk assessment scores (post vs. pre) were higher in the SG than in the WEG (*p* = 0.009). Post-discharge fall events were numerically fewer but not significantly different. There were no statistically significant between-group differences in rehospitalization.	The falling rate was 46% lower in the IG than in the CG (IRR = 0.545, 95% CI = 0.307–0.968; *p* = 0.020); the post-test mean FOF score was significantly lower in the IG than in the CG (*p* < 0.001).	Twelve allied health assistants delivered scripted education sessions to 254 patients in the IG; 97% received education within 24 h after admission. The difference in fall rates between groups was not statistically significant (IRR = 0.66, 95% CI 0.32–1.36; *p* = 0.26). There were 2.02 injurious falls per 1,000 bed days in the CG and 1.03 in the IG; nine falls (7 CG, 2 IG) required transfer to an acute facility. No adverse events were attributable to the IG intervention.
Education-related outcomes	Increased (greater reduction in FOF/FES-I in the PAPE group).	Not mentioned	Increased (fall-risk self-assessment scores in the SG).	Increased (decreased FOF in IG).	Not mentioned
Behavior related outcomes	Not mentioned	Not mentioned	No significant differences (home-environment changes).	Not mentioned	Not mentioned
Result measures	TUG; Tinetti test; FES-I.	Recurrent falls; hospital readmission; length of hospital stay; injury severity (ISS); ADL.	Fall-risk assessment scores (4 laminated color pictures); fall events; home-environment changes; all-cause rehospitalization.	Falling rate questionnaire; FES-I.	Feasibility of AHA delivery; falls per 1,000 bed days; injurious falls; falls requiring transfer to an acute medical facility.
Outcome follow-up duration	Immediate post-test only	6 months after discharge	1 week, 3 months, and 6 months after discharge.	Hospital discharge, 1 month, and 3 months after discharge.	20-week study period.
Major findings	Adding therapeutic patient education to physical activity may reduce fear of falling more than physical activity alone; no direct fall-event outcomes were assessed.	The nursing discharge planning program reduced recurrent falls, readmission, length of stay, and injury severity.	Simulation education improved recognition of home environmental fall hazards more than written handout education, but fall events and rehospitalization did not differ significantly.	SVE is effective in significantly reducing falling rate and FOF. Context-based SVE is recommended to reduce falling rate and FOF among hospitalized older people.	It is feasible and of benefit to supplement usual care with fall prevention education delivered by allied health assistants.

ADL, activities of daily living; AHA, allied health assistant; AOR, adjusted odds ratio; ARR, adjusted rate ratio; aHR, adjusted hazard ratio; CG, control group; CI, confidence interval; COM-B=Capability Opportunity Motivation Behavior; CP, control period; CPG, complete program group; DVD, digital video disk; FES-I=Falls Efficacy Scale–International; FOF, fear of falling; IADL, instrumental activities of daily living; IG, intervention group; IP, intervention period; IQR, interquartile range; IRR, incidence rate ratio; ISS=Injury Severity Score; MG, materials group; MSc, Master of Science; OR, odds ratio; PA, physical activity; PAPE, physical activity and patient education; PT, physiotherapist; RCT, randomized controlled trial; RR, relative risk; SDH=Swan Districts Hospital; SG, simulation group; SVE, simulated video education; TPE, therapeutic patient education; TUG, Timed Up and Go; WEG, written education group.

The quality of the educational programs was assessed using the metric tool developed by Heng et al. (17). This instrument evaluates the educational-design quality of interventions by focusing on pedagogical elements rather than on methodological rigor alone. The tool comprises 17 items that identify flaws in educational content and delivery that may influence participants’ learning, thereby providing a more relevant assessment for studies evaluating educational programs regardless of design. Each item is scored 1 for a positive response and 0 for a negative or uncertain response. The scores ranging from 0 to 6 indicate low quality, 7 to 12 indicate moderate quality, and 13 to 17 indicate high quality. Two researchers (Y. Y and Y. W) independently assessed the quality of the health-education programs reported in the 12 included reports; the results are presented in Table 2.

**Table 2:** The metric quality of education programs [adopted from Heng et al. (17)].

Studies included in the review		Haines et al. (14)	Hill et al. (20)	Haines et al. (23)	Hill et al. (21)	Hill et al. (5)	Naseri et al. and Hill et al. (24, 25)	Perrot et al. (29)	Dadgari et al. (27)	DeWalt et al. (28)	Valieiny et al. (26)	Morris et al. (22)
Purpose (1–4)	1. ls the purpose and rationale of the education program stated?	1	1	1	1	1	1	1	1	1	1	1
	2. Is there a clear direction to the program?	1	1	1	1	1	1	1	1	1	1	1
	3. Is there a satisfactory description of the significance of the program?	1	1	1	1	1	1	1	1	1	1	1
	4. ls the education conducted in a suitable setting?	1	1	1	1	1	1	1	1	1	1	1
Learner characteri-stics (5–6)	5. ls the program pitched toward an appropriate audience?	1	1	1	1	1	1	1	1	1	1	1
	6. ls there recognition of learner’s/ co-learner’s prior knowledge/experience?	1	1	1	1	1	1	1	1	1	1	1
Teacher characteri-stics (7–10)	7. Is there a description of who is teaching the program?	1	0	1	1	1	1	1	1	1	1	1
	8. Are the teachers qualified and/or experienced on the topic?	1	1	1	1	1	1	1	1	1	1	1
	9. Are the teachers qualified and/or experienced in teaching?	1	1	1	1	1	1	1	1	1	1	1
	10. Is training on the program offered?	1	0	1	1	1	1	1	0	0	0	1
Learning activities (11–13)	11. ls there a description of the learning activities?	1	1	1	1	1	1	1	1	1	1	1
	12. Are the learning activities suitable for supporting learners/co-learners to meet the learning objectives?	1	1	1	1	1	1	1	1	1	1	1
	13. ls there an assessment of learner’s/co-learner’s achievement of learning objectives (knowledge, skills, attitudes)?	1	1	1	1	1	1	1	1	1	1	1
Evaluation (14–17)	14. Has an evaluation been planned?	1	1	1	1	1	1	1	1	1	1	1
	15. ls the evaluation method appropriate?	1	1	1	1	1	1	1	1	1	1	1
	16. Has an evaluation been conducted?	1	1	1	1	1	1	1	1	1	1	1
	17. Are the education outcomes reported for process (learner’s/co-learner’s views on the teaching)?	1	1	1	1	1	1	1	1	1	1	1
Total (17)	0–6: Low 7–12: Moderate 13-17: High	17	15	17	17	17	17	17	16	16	16	17
		High	High	High	High	High	High	High	High	High	High	High

### 2.7 The risk of bias assessment

Two researchers (Y. Y, and Y. W) independently assessed the risk of bias using the Cochrane Risk of Bias tool (ROB 2), which was developed specifically for randomized trials (18, 19). The tool comprises five domains, each covering distinct aspects of trial design. Every domain contains signaling questions intended to elicit information pertinent to bias risk. An algorithm converts the responses into domain-level judgments, classifying each as “low risk,” “some concerns,” or “high risk.”

## 3 Results

### 3.1 Description of included reports and risk of bias

Twelve reports describing 11 trial-based evaluations of fall prevention education were included. Of these evaluations, seven were conducted in Australia (5, 14, 20–25), two in Iran (26, 27), one in the USA (28), and one in France (29). By design, six evaluations were individually randomized RCTs (23, 25–29), one was a pilot RCT (21), one was a feasibility RCT (22), and one was a stepped-wedge cluster-randomized trial (5). Two evaluations required design qualification: Haines was an education-component subgroup report (14) from the Haines targeted multifactorial RCT (30); the analyzed subgroup received one-to-one bedside education in addition to usual care and could also receive other targeted components when indicated. Hill was a DVD–workbook randomized comparison with an added non-randomized no-education control group (20). Naseri was treated as a companion behavior/process report of Hill and was not counted separately (24, 25). Among individually randomized evaluations, randomized sample sizes ranged from 30 to 1,206 participants; Hill additionally included 122 non-randomized control participants. The stepped-wedge cluster-randomized trial included 3,606 admissions.

**Figure 2:**
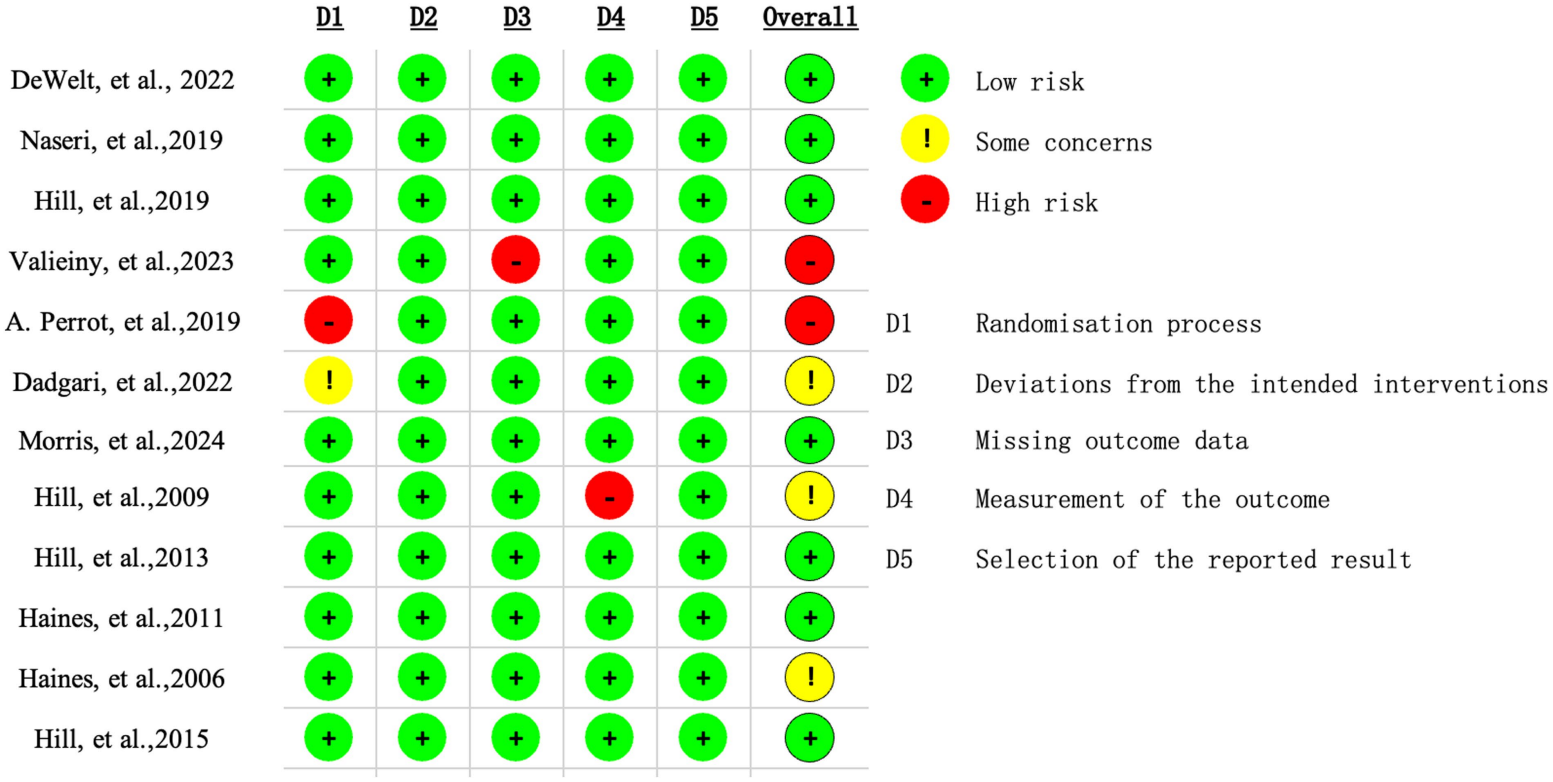
Assessments of the risk of bias of the included studies.

**Figure 3:**
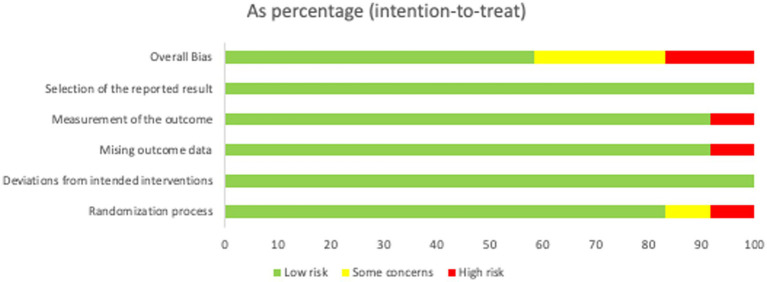
Risk of bias of the included studies by types of bias.

Risk of bias was assessed across the 11 trial-based evaluations using the revised Cochrane Risk of Bias tool (RoB 2), with Hill and Naseri treated as one evaluation. The risk-of-bias results are summarized in (Figures 2, 3) Overall, six evaluations (54.5%) were judged to have low risk of bias, three (27.3%) raised some concerns, and two (18.2%) were at high risk. Domain-level analysis showed that concerns were concentrated in three domains. For D1, the randomization process was rated low risk in nine evaluations (81.8%), whereas Perrot was rated high risk and Dadgari raised some concerns. D2, deviations from intended interventions, was rated low risk across all evaluations. For D3, missing outcome data were adequately addressed in 10 evaluations (90.9%), although Valieiny was rated high risk because of attrition. For D4, measurement of the outcome was rated low risk in 10 evaluations (90.9%), but Hill was rated high risk because unblinded assessors evaluated subjective outcomes. D5, selection of the reported result, was rated low risk across all evaluations.

### 3.2 Characteristics of the fall prevention education programs

#### 3.2.1 Health education approaches

Among the 11 FPE programs, Perrot et al. used group-based education, whereas the remaining 10 evaluations used individual-level or one-to-one education (5, 14, 20–29).

#### 3.2.2 Health education methods

Education methods included group therapeutic patient education sessions, simulated home-environment education, print materials, videos or DVDs, tailored bedside discussions or interviews, home visits, and telephone reinforcement/follow-up; the specific combinations used in each report are summarized in Table 1 (5, 14, 20–29).

#### 3.2.3 Fall prevention education content

Most reports incorporated educational material that defined falls and addressed their risk factors, causes, consequences and preventive strategies. Hill et al. incorporated modules aimed at enhancing personal beliefs related to fall prevention (20). Hill et al. included fall risk and prevention strategies for individuals discharged from the hospital with decreased physical functioning (21). In a subsequent trial, Hill et al. expanded the content to include the nature of falls, benefits of adherence, and self-efficacy enhancement, goal setting, and action planning (5). Valieiny et al. used video sequences to explain the importance, outcomes, risk factors, preventability, and prevention of falls (26). Perrot et al. devoted the first of four 1-h sessions to understanding falls, including fall history, fall definition, intrinsic and extrinsic risk factors, and describing immediate and long-term physical, psychological, social and behavioral consequences, and prevention strategies (29). Dadgari et al. opened the program with a definition of falls in older adults, emphasized prevention relevance, and differentiated intrinsic from extrinsic causes (27). Morris et al. centered on fall-risk awareness and preventive behaviors, and goal-oriented action planning (22). In the education-recommended subgroup reported by Haines et al., the program covered risk factors, mechanisms and preventive measures (14); in a subsequent RCT, Haines et al. added epidemiological data, self-reflection of individual risk, preventive strategy development, goal setting, and goal review (23).

Six reports explicitly addressed environmental safety: four focused on home or post-discharge environments and two on the hospital setting. DeWalt et al. simulated a home environment in the hospital to teach patients to recognize 14 environmental fall hazards (28). Hill et al. targeted home modification as part of individualized post-discharge fall prevention education (25). Hill et al. provided ward-based education and incorporated patient feedback about ward environmental hazards into staff support (5). Valieiny et al. used a brief video to deliver hospital-based environmental education, covering the nurses’ station, emergency exits, toilets, wall-mounted and toilet handrails, the call-bell system and bedside lighting (26). Perrot et al. dedicated one of four 1-h sessions to environmental factors, including home hazard identification and countermeasures (29). Dadgari et al. conducted a home-visit session to assess each participant’s living environment and recommend modifications (27).

#### 3.2.4 Follow-up and reinforcement of fall prevention education

Among the included reports, six described protocol-specified repeated education, reinforcement, or post-education contact. In-hospital reinforcement or repeated educational contact was reported by Haines et al. (2006), Haines et al. (2011), Hill et al. (2015), and Morris et al. (2024), whereas post-discharge telephone reinforcement was used by Hill et al. (2013) and Hill et al. (2019). Haines et al. delivered repeated bedside education sessions during hospitalization (14), and Haines et al. employed individualized bedside follow-up during the first study week, delivered by trained physical therapists (23). Hill et al. used individually tailored follow-up sessions with ward visits and staff feedback during the intervention period (5). Hill et al. carried out a single telephone call 2 weeks after discharge to reinforce the education (21). Hill et al. provided three monthly coaching calls after discharge from the same therapist to reinforce behavior change, provide feedback, identify barriers, and revise fall prevention action plans (25). Morris et al. scheduled brief conversations during admission and an individual bedside follow-up on day 5 after initial in-hospital education (22). Valieiny et al. assessed patients with the Falls Efficacy Scale-International at discharge and at 1 and 3 months after discharge; this represented outcome follow-up rather than additional education reinforcement (26).

#### 3.2.5 Duration of fall prevention education

Among reports with sufficient duration data, reported education contact ranged from a 14-min DVD or 15-min simulated-video intervention to four 1-h therapeutic education sessions. Most reports described session-level or initial education contact of ≤1 h, whereas multi-session programs could exceed 1 h in total. Home-visit time, post-discharge telephone reinforcement, and outcome follow-up periods were excluded from this duration summary.

### 3.3 Principles and theoretical framework of fall prevention education design

Four reports used the Health Belief Model (HBM): Hill et al., Haines et al., Hill et al., and Hill et al. (5, 20, 21, 23). Originally developed in the 1950s, the HBM explains health behavior through perceived susceptibility, perceived severity, perceived benefits, perceived barriers, self-efficacy, and cues to action (31, 32).

The post-discharge education program reported by Hill et al. was designed according to the COM-B model, which conceptualizes behavior change as the interaction among capability, opportunity and motivation (25, 33).

Two reports used simulation-based education (SBE), grounded in experiential learning theory, to enhance learner engagement: DeWalt et al. and Valieiny et al. (26, 28, 34). DeWalt et al. implemented SBE incorporating realistic visual displays, demonstrations, repetitive practice, and role-play to improve critical thinking and self-confidence (28). Valieiny et al. provided simulation-based video education that allowed repeated exposure, delivered standardized content delivery, time and cost saving, increased motivation and knowledge retention, and training in scenarios that would be hazardous or impractical in real clinical settings (26).

Two reports used motivationally oriented approaches: Morris et al. used motivational interviewing, and Haines et al. applied Protection Motivation Theory (14, 22). Morris et al. used motivational interviewing (MI), as described by Miller and Rollnick (22, 35). MI emphasizes empathy and non-directive communication, elicits intrinsic motivation, and facilitates change by helping individuals explore discrepancies between their behavior and goals (35). Haines et al. applied the Protection Motivation Theory (PMT), together with threat appraisal and goal setting/review (14). PMT explains how persuasive communication influences behavior through cognitive processes that endorse or resist recommended actions (36). Patient goal setting, goal review, action planning, or goal-oriented strategy planning was reported (5, 14, 21–23, 25).

Perrot et al. introduced therapeutic patient education (TPE), a structured, person-centered learning process that mobilizes patients’ internal resources and supports self-management (29). TPE is delivered by professionally trained healthcare providers and tailored to patient’s circumstances (37). It aims to help patients acquire specific or generic medical knowledge and self-management skills, including preventive service use, healthy lifestyles, and management of illness or accidents at home (37).

Dadgari et al. implemented a discharge-planning program derived from the nursing process (27). This framework comprises assessment, problem identification, care-plan formulation, implementation, and evaluation, and coordinates healthcare resources with patient needs to support transition from hospital to home or community settings (38). Structured discharge education has also been associated with clearer discharge instructions in a quality-improvement study (39).

### 3.4 Assessment of fall prevention education program quality

The quality of the fall prevention education programs was assessed using the Modified Quality Assessment Scale for Educational Programs (17), after excluding items specific to clinical physician training. The scale evaluates core educational elements, including objectives and implementation environment, characteristics of learners and instructors, learning-activity design, and program effectiveness (17).

The 11 assessed fall prevention education programs were rated as high quality, with scores ranging from 15 to 17 on a 17-point scale. Maximum scores were achieved for objectives, learner characteristics, learning activities, and evaluation. Points were deducted primarily in the instructor-characteristics domain: implementer training was not reported in four reports—Hill et al. (2009), Dadgari et al. (2022), DeWalt et al. (2023), and Valieiny et al. (2023)—and Hill et al. (2009) also did not clearly identify the personnel delivering the education (20, 26–28).

### 3.5 Evaluation of the efficacy of fall prevention education for older inpatients

#### 3.5.1 Fall-related outcome measures

Nine of the 11 trial-based evaluations assessed fall-related outcomes, including fall rate/incidence, injurious falls, faller proportion, fractures, and injury severity. Reductions in at least one fall-related measure were reported by Haines et al. (2006), Hill et al. (2013), Hill et al. (2015), Dadgari et al. (2022), and Valieiny et al. (2023), whereas Haines et al. (2011) reported a subgroup effect among cognitively intact patients only (5, 14, 21, 23, 26, 27). Haines et al. reported a lower incidence of falls in the intervention group than in the control group (8.2 vs. 16.0 falls/1000 participant-days, *p* = 0.007), although the proportion of fallers did not differ significantly (14). Hill et al. reported a lower fall rate after discharge in the intervention group than in the control group (5.4 vs. 18.7 falls/1000 person-days, *p* = 0.05) (21). Hill et al. reported lower rates of falls and injurious falls during the intervention period than during the control period (falls: 7.80 vs. 13.78/1000 patient-days, ARR = 0.60, 95% CI 0.42–0.94, *p* = 0.003; injurious falls: 2.63 vs. 4.75/1000 patient-days, ARR = 0.65, 95% CI 0.42–0.88, *p* = 0.006) (5). Dadgari et al. reported lower falls and injury severity in the intervention group than in the control group (*p* < 0.049 and *p* < 0.001, respectively) (27). Valieiny et al. reported a lower falling rate in the intervention group than in the control group (IRR = 0.545, 95% CI = 0.307–0.968; *p* = 0.020) (26).

Haines et al. (2011) found no significant difference in falls per 1,000 patient-days in the total sample, but reported a significant interaction between intervention and cognitive impairment. Among cognitively intact patients, falls were less frequent in the complete program group than in the materials-only group and usual-care group (4.01 vs. 8.18 and 8.72 falls/1000 patient-days, complete program group vs. materials-only group, adjusted hazard ratio, 0.51, 95% CI, 0.28–0.93, complete program group vs. usual-care group, adjusted hazard ratio, 0.43, 95% CI, 0.24–0.78). Among cognitively impaired patients, injurious falls were higher in the complete program group than in the usual-care group (7.49 vs. 2.89 injurious falls/1000 patient-days) (23).

Several evaluations did not show statistically significant effects on fall-event outcomes. Hill et al. reported no significant between-group differences in 6-month fall rate, injurious fall rate, or faller proportion after discharge (25). DeWalt et al. reported fewer post-discharge fall events numerically, but the between-group difference was not statistically significant (28). Morris et al. found no significant difference in fall rates between groups (IRR = 0.66, 95% CI 0.32–1.36; *p* = 0.26) (22).

#### 3.5.2 Education-related outcome measures

Education-related outcomes included participants’ perceptions of the education program, fall-prevention knowledge, fall-risk awareness or self-assessment, self-perceived fall risk, confidence and motivation, and fear of falling. These outcomes were reported (14, 20, 21, 26, 28, 29). Haines et al. reported positive program-evaluation responses, including that participants found the materials understandable and gained new information (14). Hill et al. reported higher fall-prevention knowledge in the DVD and workbook groups than in the no-education quasi-control group (*p* < 0.001), and higher self-perceived risk of falling, confidence, and motivation in the DVD group than in the workbook group (*p* = 0.04, *p* = 0.03, and *p* = 0.04, respectively) (20). Hill et al. reported that the intervention group was significantly more knowledgeable, confident, and motivated to engage in fall-prevention strategies than the control group (21). DeWalt et al. reported higher fall-risk post-assessment scores in the simulation group than in the written-education group (*p* = 0.022), with greater pre–post changes in fall-risk assessment scores in the simulation group (*p* = 0.009) (28). Perrot et al. reported a significant group × time interaction for the Falls Efficacy Scale-International, indicating a greater reduction in fear of falling in the therapeutic patient education plus physical activity group (29). Valieiny et al. reported a lower post-test mean fear-of-falling score in the intervention group than in the control group (*p* < 0.001) (26).

#### 3.5.3 Behavior-related outcome measures

Behavior-related outcomes included risk-reducing actions, seeking or waiting for assistance, identifying environmental hazards, using assistive devices, wearing safe footwear or clothing, increasing physical activity, seeking assistance for ADL/IADL, safely resuming functional activities, participating in a home exercise program, making home modifications, and engagement in post-discharge fall-prevention strategies. These outcomes were reported in Haines et al. (2006), Haines et al. (2011), Hill et al. (2013), DeWalt et al. (2023), and Naseri et al. (2019), the companion behavior report of the Hill et al. (2019) trial (14, 21, 23, 24, 28). Haines et al. reported that participants modified their actions to reduce their risk of falling (14). Haines et al. reported increased use of selected risk-reducing behaviors, including seeking or waiting for assistance, identifying environmental hazards, using assistive devices, wearing safe footwear or clothing, and doing more exercise (23). Hill et al. reported greater engagement in selected post-discharge strategies; participants in the intervention group were more likely to plan how to safely restart functional activities than those in the control group (AOR = 3.80, 95% CI 1.07–13.52; *p* = 0.04), whereas the difference in completing a home exercise program was not statistically significant (AOR = 2.76, 95% CI 0.72–10.50; *p* = 0.14) (21). DeWalt et al. found no significant between-group difference in home-environment changes (28), and Naseri et al. reported no significant differences in engagement in ADL/IADL assistance, home-hazard modification, or exercise after discharge (24).

#### 3.5.4 Related health-service outcome measures

Related health-service outcomes were reported in three trial-based evaluations and included readmission or rehospitalization and length of stay. Dadgari et al. reported a lower readmission rate (*p* = 0.014) and shorter length of hospital stay (*p* = 0.018) in the intervention group than in the control group (27). DeWalt et al. found no statistically significant between-group difference in rehospitalization (28). Hill et al. also found no significant difference in length of stay between the intervention and control periods (5).

### 3.6 Providers of fall prevention education for older inpatients

Across the 11 trial-based evaluations, fall prevention education was delivered by therapists in five evaluations, including physiotherapists in Haines et al. (2011), Hill et al. (2013), Hill et al. (2015), and Hill et al. (2019)/Naseri et al. (2019), and an occupational therapist in Haines et al. (2006) (5, 14, 21, 23–25). Three evaluations involved nurses or a nurse investigator as providers (26–28). The remaining evaluations were delivered by a trained MSc sport science student (Perrot et al., 2019), allied health assistants (Morris et al., 2024), or investigators whose professional background was not specified (Hill et al., 2009) (20, 22, 29). Overall, therapists—particularly physiotherapists—were the most frequently reported providers, while nurses also delivered fall prevention education in several evaluations.

## 4 Discussion

### 4.1 Principal findings across outcome domains

Across the 12 reports describing 11 trial-based evaluations, fall prevention education was assessed primarily through fall-related outcomes, including falls, fall rates, faller proportion, injurious falls, fractures, and injury severity, with fewer evaluations measuring education-related, behavior-related, and related health-service outcomes. Haines et al. (2006), Hill et al. (2013), Hill et al. (2015), Dadgari et al. (2022), and Valieiny et al. (2023) reported reductions in at least one fall-related measure, while Haines et al. (2011) reported benefit only among cognitively intact patients and a potential adverse signal for injurious falls among cognitively impaired patients (5, 14, 21, 23, 26, 27). By contrast, Hill et al. (2019), DeWalt et al. (2023), and Morris et al. (2024) did not show statistically significant fall-event effects (22, 25, 28). Education-related outcomes generally moved in a favorable direction when measured, as reported by Haines et al. (2006), Hill et al. (2009), Hill et al. (2013), Perrot et al. (2019), DeWalt et al. (2023), and Valieiny et al. (2023), but these intermediate outcomes were not consistently linked to sustained behavior change or fall reduction (14, 20, 21, 26, 28, 29). Behavior-related findings were also mixed: selected risk-reducing actions improved in Haines et al. (2006), Haines et al. (2011), and Hill et al. (2013), whereas DeWalt et al. (2023) and Naseri et al. (2019), as the companion behavior report of the Hill et al. (2019) trial, found no significant between-group differences in home-environment changes or post-discharge strategy engagement (14, 21, 23, 24, 28). Health-service outcomes were reported in only a small number of evaluations and showed mixed findings: Dadgari et al. (2022) reported reductions in readmission and length of stay, whereas DeWalt et al. (2023) and Hill et al. (2015) found no significant differences in rehospitalization or length of stay (5, 27, 28). Future trials should pre-specify linked education, behavior, fall-event, and health-service outcomes to clarify whether gains in knowledge, awareness, or motivation translate into sustained risk-reducing behavior and clinically meaningful reductions in falls.

### 4.2 Interpretation of outcome inconsistency

The inconsistent effects on fall-related outcomes may partly reflect differences in prevention targets, education settings, and follow-up periods across the included evaluations. In-hospital programs such as Haines et al. (2006), Haines et al. (2011), Hill et al. (2015), Valieiny et al. (2023), and Morris et al. (2024) were delivered during admission, whereas Hill et al. (2013), Hill et al. (2019), Dadgari et al. (2022), and DeWalt et al. (2023) focused partly or mainly on post-discharge or home-related fall prevention (5, 14, 21–23, 25–28). These settings differ in the degree to which patients can translate education into action, because inpatient behavior is shaped by ward routines, staff responsiveness, and environmental controls, whereas post-discharge behavior depends more on functional recovery, home support, and sustained self-management.

Intervention intensity and reinforcement may also have contributed to the mixed findings. Programs with repeated contact, tailored discussion, goal setting, or staff feedback, such as Haines et al. (2006), Haines et al. (2011), Hill et al. (2015), Hill et al. (2019), and Morris et al. (2024), differed from one-off or brief education approaches, making it difficult to separate the effect of educational content from the effect of delivery intensity and implementation support (5, 14, 22, 23, 25). Patient characteristics are another likely source of heterogeneity, particularly cognitive status. Haines et al. found no significant effect in the total sample but reported fewer falls among cognitively intact patients and a potential adverse signal for injurious falls among cognitively impaired patients, suggesting that fall prevention education may require adaptation for patients with impaired cognition rather than uniform delivery across all older inpatients (23).

The pathway from education-related outcomes to behavior change and fall reduction also remains uncertain. Improvements in knowledge, confidence, motivation, fall-risk awareness, or fear of falling were reported in several evaluations, but behavior-related outcomes were measured less consistently, and the companion behavior report by Naseri et al. did not show greater post-discharge engagement in fall prevention strategies in the Hill et al. trial (20, 21, 24, 26, 28, 29). This suggests that fall prevention education may improve proximal cognitive or affective outcomes without necessarily producing sustained behavior change or fall-event reductions, especially after discharge.

Methodological differences further limit direct interpretation of between-study inconsistency. The evidence base included conventional individually randomized RCTs, a pilot RCT, a feasibility RCT, a stepped-wedge cluster-randomized trial, an education-component subgroup analysis from a parent multifactorial RCT, and a DVD–workbook randomized comparison with an additional non-randomized control group. Therefore, the direction of findings should be interpreted alongside study design, sample size, risk of bias, intervention fidelity, and outcome follow-up rather than as evidence that fall prevention education has a single uniform effect.

### 4.3 Program characteristics and delivery features

The educational content of the included fall prevention education programs clustered around three recurring areas. First, fall risk, fall-related consequences, risk factors, preventive strategies, and benefits of adherence were addressed in programs reported (5, 14, 20–23, 26, 27, 29). Second, environmental safety was incorporated in programs targeting either hospital or home hazards (5, 25–29). Third, post-discharge risk management was emphasized in Hill et al. (2013), Hill et al. (2019), Naseri et al. (2019), Dadgari et al. (2022), and DeWalt et al. (2023), including home modification, functional activity, exercise, assistance with daily activities, and safe performance of activities after discharge (21, 24, 25, 27, 28).

These content areas are broadly consistent with guideline recommendations that at-risk patients, and when appropriate their families, should receive information on fall risk, fall prevention, and relevant interventions (40). The same guideline also supports individualized, multi-component exercise and balance training for adults at risk of falls; however, this evidence relates to active fall-prevention interventions rather than to education content alone (40). Medication-related fall risk was not directly evaluated as an education component in the included programs. Given that Jehu et al. reported an association between medication-related factors and recurrent falls in older adults, medication-safety messages may be considered in future fall prevention education programs, particularly when education is integrated with multidisciplinary fall-risk assessment; however, this review does not provide direct evidence that adding medication content improves fall-related outcomes (41).

Among the 11 fall prevention education programs, 10 used individual-level delivery, whereas Perrot et al. used group-based therapeutic patient education. Individual-level delivery was reported (5, 14, 20–29). This pattern supports the relevance of individualized or tailored education for older inpatients, because fall risk, functional limitations, discharge destination, and environmental hazards vary across patients. This delivery orientation is also supported by discharge-planning evidence showing that participation in individualized decision-making and effective information exchange explained 71% of the variance in satisfaction with discharge plans and continuity of care among older inpatients; tailored health education was also reported to improve functional status, including balance and activity safety (42). However, the available fall prevention education studies did not directly compare individual- and group-based delivery for fall-event reduction.

Among programs with sufficient duration information, session-level or initial education contact generally lasted no more than 1 h, although multi-session programs and post-discharge reinforcement could exceed this duration. Brief or one-off education was reported by Hill et al. (2009) and DeWalt et al. (2023), short simulated-video education was used by Valieiny et al. (2023), and repeated sessions, tailored follow-up, home visits, or reinforcement were reported (5, 14, 20–29). These findings highlight a feasibility trade-off: shorter education may be more compatible with inpatient workflow, whereas repeated reinforcement or tailored follow-up may be needed to support behavior change. Duration findings should be interpreted cautiously because studies varied in whether they counted only initial education contact, repeated sessions, home visits, or post-discharge reinforcement. The barriers reported by Pueyo-Garrigues et al., including insufficient training, inadequate time, and excessive workload, further support the need to design concise, trainable, and workflow-compatible fall prevention education programs (43).

### 4.4 Theoretical basis and program quality

From an ecological perspective, health-education models can be grouped into individual capability, interpersonal relationships, and environmental contexts. Individual-capability models include the Health Belief Model (HBM), Transtheoretical Model of Change, Theory of Planned Behavior, and related cognitive-behavioral models; interpersonal and environmental-context models include Social Cognitive Theory, Communication Theory, and Diffusion of Innovations Theory. Rimer et al. noted that evidence-based, theory-driven interventions are most likely to demonstrate effectiveness in empirical research. Combining theory-based interventions with rigorous evaluation clarifies why programs succeed or fail under specific conditions (44).

In this systematic review, HBM was the most frequently used theoretical model and was applied (5, 20, 21, 23). This predominance is likely related to the way fall prevention education for hospitalized older adults is commonly conceptualized. Such interventions typically target patient-level risk perception and safety behavior, aiming to strengthen awareness of personal susceptibility to falls and the seriousness of fall-related harm, increase perceived benefits of preventive actions, and reduce perceived barriers to seeking assistance, using assistive devices, and adhering to safety advice. In this respect, HBM provides a close conceptual fit for educational interventions designed to improve knowledge, risk awareness, and adherence-related behaviors. However, HBM is primarily centered on individual cognition and pays less attention to contextual determinants, such as ward environment, staff responsiveness, care processes, and post-discharge support. In addition, emphasizing severity without corresponding supportive strategies may risk increasing fear of falling.

The 11 assessed fall prevention education programs received high scores in the quality assessment, with scores ranging from 15 to 17 on a 17-point scale. However, implementer training was not reported in four reports—Hill et al. (2009), Dadgari et al. (2022), DeWalt et al. (2023), and Valieiny et al. (2023)—and Hill et al. (2009) also did not clearly identify the personnel delivering the education (20, 26–28). Equipping education providers with relevant knowledge and skills may enhance health-education efficacy and support more consistent program implementation. In a cross-sectional study, Pueyo-Garrigues et al. reported that nurses directly involved in patient care had insufficient health-education knowledge, with inadequate training being the main barrier (71.4%) (43). Because nurses delivered fall prevention education in several included evaluations and commonly participate in hospital patient education, their training and competencies warrant attention (26–28). Pueyo-Garrigues et al. advocated comprehensive education and training programs, together with a hospital-wide culture and infrastructure for health promotion, to enhance the knowledge, skills, and personal qualities related to health education (43).

### 4.5 Implications for practice and research

In clinical practice, fall prevention education is likely to be more sustainable when embedded within a multidisciplinary workflow. Nurses’ continuous bedside presence may facilitate repeated risk communication, safety reminders, and discharge preparation, whereas physiotherapists and occupational therapists contribute expertise in mobility, exercise, assistive devices, environmental hazards, and post-discharge functional recovery. The feasibility study by Morris et al. (2024) also suggests that trained allied health assistants, under professional supervision and with clear escalation pathways, may support timely delivery of scripted fall prevention education within 48 h of admission (22). These findings indicate that implementation models should clarify how educational responsibilities are shared across professional groups, how educators are trained and supervised, and how education is reinforced throughout admission, ward care, and discharge.

Further research is needed to evaluate different delivery models as implementation strategies for fall prevention education. Future studies should specify educator selection, training, supervision, fidelity monitoring, and professional role boundaries, and should examine whether nurse-led, therapist-led, assistant-supported, or multidisciplinary delivery influences education-related, behavior-related, fall-event, and related health-service outcomes. Such evidence would help identify scalable models for integrating fall prevention education into routine hospital care.

### 4.6 Strengths and limitations

A strength of this review is that it examined not only fall-event outcomes but also the theoretical basis, educational content, delivery features, providers, and program quality of fall prevention education programs. This approach allowed a more detailed interpretation of why education-related outcomes, behavior-related outcomes, and fall-event outcomes were not always aligned across the included evaluations.

First, sample size and analytic denominator varied across the 11 trial-based evaluations. Three evaluations enrolled fewer than 100 participants (Hill et al., 2013; Perrot et al., 2019; DeWalt et al., 2023), six enrolled 100 to fewer than 1,000 participants or used comparable trial denominators (Haines et al., 2006; Hill et al., 2009; Hill et al., 2019/Naseri et al., 2019; Dadgari et al., 2022; Valieiny et al., 2023; Morris et al., 2024), and two included at least 1,000 participants or admissions (Haines et al., 2011; Hill et al., 2015). Smaller evaluations may produce less precise and more variable estimates; therefore, positive findings from smaller studies should be interpreted cautiously in the narrative synthesis. Risk-of-bias concerns also reduced confidence in the evidence, with two evaluations rated as high risk of bias and three as having some concerns.

Substantial clinical and methodological heterogeneity also precluded meta-analysis. The included evaluations differed in prevention target, education setting, study design, outcome follow-up, intervention content, delivery method, education duration or reinforcement, provider type, and theoretical or pedagogical basis. These differences indicate that the included programs were not sufficiently comparable for a pooled estimate of effect. Four reports used the Health Belief Model, but the extent to which theoretical constructs were translated into education content, delivery procedures, and outcome measures varied across programs. Similarly, the two simulation-based education programs differed in focus, with DeWalt et al. (2023) emphasizing home-environment hazards and Valieiny et al. emphasizing hospital-based fall prevention scenarios. This heterogeneity made meta-analysis inappropriate, a concern also highlighted by Almas et al. (45).

A further limitation is that the review focused on randomized or trial-based evaluations. This focus may have excluded relevant non-randomized, observational, mixed-methods, and qualitative evidence, thereby narrowing insight into implementation processes, contextual influences, educator perspectives, and patient experiences. Future evidence syntheses could incorporate a broader range of study designs to complement trial-based evidence and provide a more comprehensive understanding of the effectiveness, feasibility, and applicability of fall prevention education for older adults.

## 5 Conclusion

This systematic review of 12 reports describing 11 trial-based evaluations suggests that fall prevention education for hospitalized older adults and those transitioning home after discharge may improve selected fall-related, education-related, and behavior-related outcomes, but effects were inconsistent across outcome domains. Gains in knowledge, risk awareness, confidence, motivation, or fear of falling were not consistently linked to sustained risk-reducing behavior or fall-event reduction. Many programs were theory-informed and the assessed fall prevention education programs were rated as high quality, but the depth of theoretical application, reporting of provider training, and linkage between theoretical constructs and outcome measures remained uneven. Although nurses or nurse investigators delivered fall prevention education in several evaluations, therapists were the most frequently reported providers, and no included evaluation directly compared outcomes by provider discipline. Future fall prevention education programs should be theory-informed, tailored to patient risk and care setting, and evaluated using rigorous designs with linked education-related, behavior-related, fall-event, and health-service outcomes. Further research should compare nurse-led, therapist-led, assistant-supported, and multidisciplinary delivery models, including nurse-delivered or nurse-reinforced education, to inform role-specific implementation decisions.

## Data Availability

Publicly available datasets were analyzed in this study. This data can be found at: data were extracted from various studies and summarized in a table. The table has been presented in the article.
