# Supplementary material for: Fall prevention education among older hospital inpatients: a systematic review
Source: Front Public Health. 2026 Jul 8;14:1829617. doi: 10.3389/fpubh.2026.1829617 (PMC13388160; doi:10.3389/fpubh.2026.1829617)
Supplement: Supplementary file 1 [file Supplementary_file_1.docx]

Appendix A. Search strategies
